# Uma Manifestação Pouco Comum de Rejeição

**DOI:** 10.36660/abc.20210671

**Published:** 2022-05-04

**Authors:** Carlos Xavier Correia de Resende, Pedro Grilo Diogo, Sandra Amorim, Gonçalo Pestana, José Pinheiro Torres, Filipe Macedo

**Affiliations:** 1 Centro Hospitalar Universitário de São Joao Porto Portugal Centro Hospitalar Universitário de São Joao, Porto – Portugal

**Keywords:** Insuficiência Cardíaca, Transplante Cardíaco, Pesquisa, Rejeição de Enxertos, Diagnóstico por Imagem, Tratamento Farmacológico, Bloqueio Cardíaco, Arritmias Cardíacas, Vasoespasmo Coronário

## Introdução

O transplante cardíaco ortotópico é o tratamento atual de escolha para pacientes selecionados com insuficiência cardíaca em estágio terminal.^1^ Com o aprimoramento das técnicas cirúrgicas e a eficiência dos novos tratamentos imunossupressores, a sobrevida em curto prazo melhorou acentuadamente ao longo dos anos.^2^ No entanto, esses pacientes ainda sofrem comorbidades importantes causadas por complicações crônicas do transplante, como rejeição, doença vascular do enxerto (DVE) e malignidade. Distúrbios do ritmo são comuns em pacientes transplantados cardíacos e, em algumas circunstâncias, podem ser a primeira manifestação clínica de rejeição.^3^ O vasoespasmo coronariano foi recentemente associado à rejeição aguda e DVE,^4^ mas os mecanismos subjacentes a esse fenômeno ainda são especulativos.

Apresentamos um caso clínico de rejeição aguda a transplante cardíaco, manifestada por vasoespasmo coronariano e distúrbio de ritmo avançado.

### Apresentação do caso

Um homem de 55 anos com insuficiência cardíaca isquêmica em estágio final foi submetido a transplante cardíaco ortotópico em março de 2019. A biópsia endomiocárdica (BEM) realizada no primeiro ano de seguimento mostrou rejeição celular leve grau 1R (ISHLT) em três amostras e rejeição moderada (2R) em duas amostras, tratadas com umas doses aumentadas de corticosteroides orais. Na última consulta ambulatorial, o paciente apresentava função ventricular esquerda normal, sem rejeição humoral ou celular identificada na última BEM (realizada 4 meses antes da hospitalização). Suas amostras de sangue mostraram níveis infraterapêuticos de ciclosporina (94,2 ng/mL), levando a um aumento da dose ambulatorial. Ele também foi medicado com micofenolato de mofetil (MMF) (2g/dia), prednisolona (5mg), atorvastatina, aspirina, cotrimoxazol e antidiabéticos orais.

Um ano e cinco meses após o transplante, foi atendido em nosso serviço de emergência com alteração súbita do estado de consciência. Ele tinha um escore de 11 na Escala de Coma de Glasgow e estava hemodinamicamente estável. O exame físico, eletrocardiograma, tomografia computadorizada do crânio e ecocardiograma transtorácico (ETT) foram normais. As amostras de sangue revelaram anemia leve (10 g/dL) e níveis infraterapêuticos de ciclosporina (92 ng/mL), mas sem elevação dos marcadores inflamatórios; a toxicologia negativa para álcool ou drogas de abuso. O paciente não colaborou durante o eletroencefalograma e devido à sua acentuada agitação psicomotora e confusão foi sedado e intubado. A punção lombar foi realizada e não mostrou sinais de infecção no líquido cefalorraquidiano. A ciclosporina foi substituída por tacrolimus, considerando a possibilidade de síndrome de encefalopatia posterior reversível.

**Figura 1:**
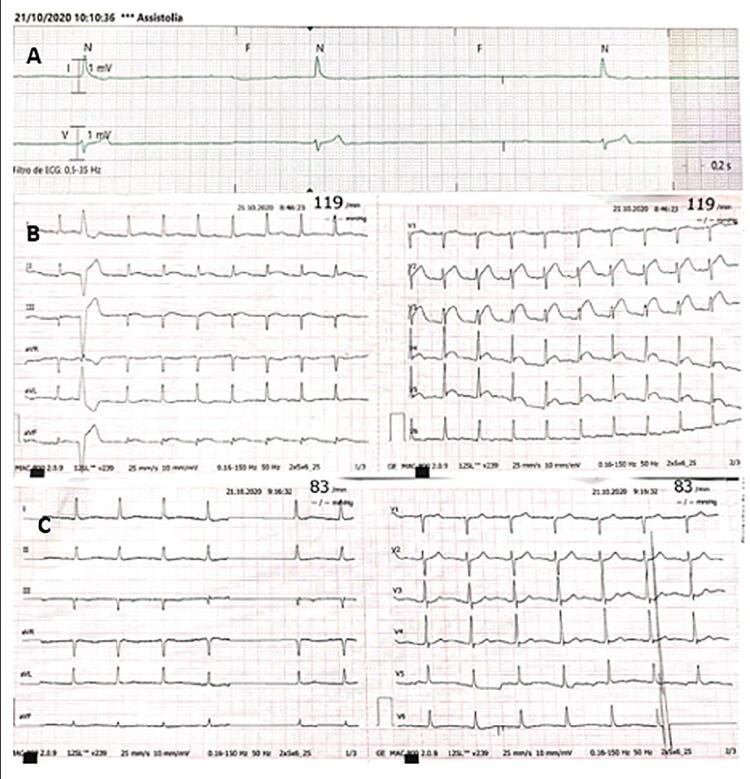
– A) Bloqueio atrioventricular de terceiro grau antes da parada cardíaca; B) ECG após RCE mostrando supradesnivelamento do segmento ST em V2-V5, extrassístole isolada; C) ECG normal 10 minutos após o evento.

O paciente foi hospitalizado na unidade de terapia intensiva e, no segundo dia de internação, apresentou parada cardíaca súbita com retorno da circulação espontânea (RCE) após dois ciclos de suporte avançado de vida. O eletrocardiograma após RCE ( Figura 1B ) registrou supradesnivelamento do segmento ST em V2-V5. Um segundo ECG 10 minutos após o evento foi normal ( Figura 1C ). Após análise do monitoramento por telemetria, foi possível identificar bloqueio cardíaco completo antes da parada cardíaca ( Figura 1A ). No mesmo dia, um segundo episódio de bloqueio cardíaco completo foi documentado, com deterioração hemodinâmica e um marcapasso temporário foi implantado.

**Figura 2:**
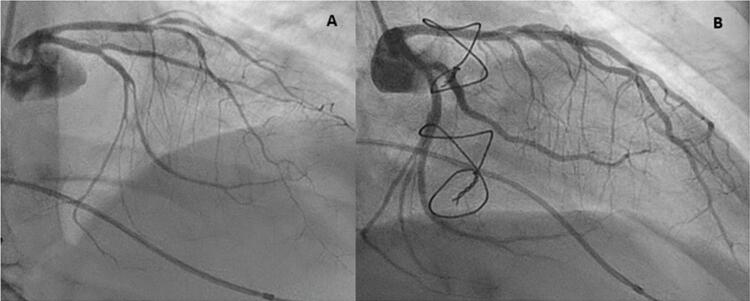
– A) Coronariografia mostrando vasoespasmo acentuadamente difuso das artérias descendente anterior e circunflexa. B) Resolução completa do vasoespasmo após ingestão de nitrato.

A cineangiocoronariografia foi realizada no dia seguinte, mostrando vasoespasmo acentuadamente difuso das artérias descendente anterior e circunflexa, que se resolveu com a administração de nitrato intracoronário ( Figura 2 A e B ). Uma estenose moderada foi identificada nas artérias descendente anterior esquerda proximal e primeira diagonal. Uma biópsia endomiocárdica também foi realizada.

**Figura 3:**
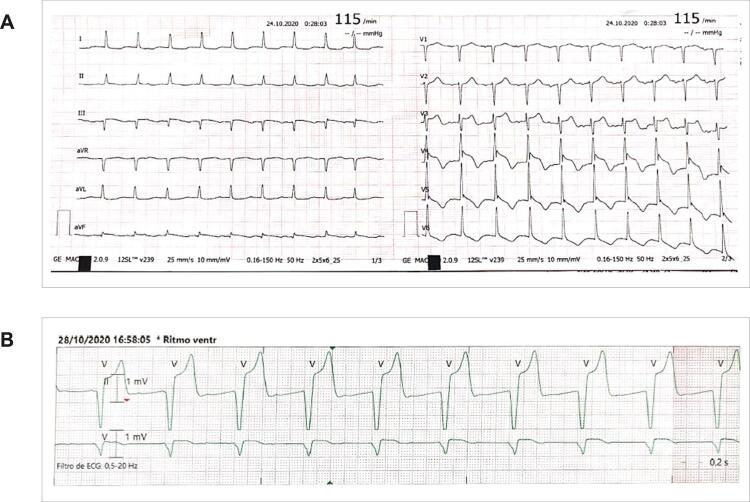
– A) Alterações transitórias de ST-T; B) Estimulação esporádica de marcapasso temporário.

Nos dias subsequentes, um novo episódio de parada cardíaca foi precedido por taquicardia ventricular, novamente com registro de alterações transitórias de supradesnivelamento do segmento ST após RCE ( Figura 3A ). A estimulação esporádica do marcapasso temporário também foi documentada ( Figura 3B ). Diante da suspeita de episódios de vasoespasmo coronariano, foi iniciada a administração de bloqueadores de canais de cálcio (BCC) e nitrato.

Os resultados da biópsia endomiocárdica mostraram rejeição celular moderada 2R (ISHLT) e rejeição humoral positiva para C4d, com posterior identificação de anticorpos anti-HLA classe II específicos do doador (HLA-DR53). O paciente foi, portanto, tratado com pulsos de metilprednisolona intravenosa e permaneceu clinicamente estável. Com a intensificação do tratamento imunossupressor, BCC e nitrato, o paciente ficou hemodinamicamente estável, sem novas alterações súbitas de ritmo ou no ECG. O diagnóstico de rejeição aguda do enxerto causando vasoespasmo coronariano e distúrbios avançados do ritmo foi considerado.

Duas semanas após o tratamento, a biópsia endomiocárdica foi repetida mostrando rejeição celular leve 1R (ISHLT) e sem sinais de rejeição humoral. Devido à resposta clínica e histológica, nenhum tratamento farmacológico adicional foi iniciado. Um cardioversor desfibrilador foi implantado sem intercorrências.

Durante a hospitalização, o paciente sofreu múltiplas infecções nosocomiais, necessitando de ventilação mecânica em duas ocasiões diferentes devido a pneumonia grave. Por causa da miopatia grave causada por hospitalização prolongada, ele recebeu alta para um centro de reabilitação e permanece estável.

## Discussão

Desde o primeiro transplante de coração humano realizado pelo Dr. Christian Barnard em dezembro de 1967,^5^ várias melhorias foram observadas ao longo dos anos em relação ao procedimento e principalmente ao manejo pós-operatório. A introdução da terapia de imunossupressão em 1980 foi um marco, permitindo uma melhora significativa nas taxas de sobrevida precoce.^6^ No entanto, a rejeição continua sendo uma das principais causas de morte após o transplante.^7^ Como os sintomas de rejeição geralmente são inespecíficos, uma alta suspeita clínica é fundamental para a detecção e tratamento imediatos. Nesse contexto, as BEMs seriadas continuam sendo a pedra angular para o diagnóstico de rejeição.

Os distúrbios do ritmo são comuns em pacientes transplantados cardíacos. No entanto, com a implementação mundial da abordagem bicaval que preserva o átrio direito e o nó sinusal, o número de eventos pós-operatórios de bradiarritmia com necessidade de marcapasso definitivo foi significativamente reduzido.^8^ A bradicardia tardia após o transplante cardíaco tem sido associada a episódios de rejeição aguda^9^ e DVE, e esta última possivelmente pode levar à isquemia do nó sinusal.^10^ Isso ressalta a importância de lembrar que, em pacientes que apresentam bradicardia ou bloqueio cardíaco tardio após o transplante, a possibilidade de rejeição aguda e DVE deve ser investigada através de ecocardiografia, BE e cinecoronariografia.^11^ Nessas circunstâncias, o implante de marcapasso é insuficiente para um tratamento eficaz até que a causa subjacente da bradiarritmia possa ser tratada.

O espasmo de artéria coronária (EAC) é uma doença subdiagnosticada, principalmente em pacientes transplantados cardíacos. Essa condição era considerada rara; porém, evidências recentes mostram uma prevalência significativa de EAC na cineangiocoronariografia de rotina nesses pacientes.^12^ Boffa et al.,^13^ documentaram vasoespasmo coronariano em 12 pacientes (5% da população estudada) após transplante cardíaco em um seguimento de 5 anos. Durante o seguimento, 80% dos pacientes desenvolveram estenose orgânica e 50% dos pacientes com múltiplos vasoespasmos morreram. Esses dados reforçam a hipótese de que o EAC após o transplante cardíaco pode ser um marcador de mau prognóstico e manifestação precoce da DVE.

A etiologia e fisiopatologia do EAC em pacientes transplantados cardíacos permanece especulativa, mas vários mecanismos têm sido relatados: sistema nervoso autônomo anormal, disfunção endotelial, hiperatividade da musculatura lisa coronariana e inflamação do componente perivascular.^14^ Uma das causas presumidas do EAC é o consumo de cocaína, devido à estimulação adrenérgica direta das artérias coronárias.^15^ Particularmente no contexto de episódios de EAC que se apresentam precocemente após o transplante, um histórico médico cuidadoso do doador e receptor do coração pode ajudar a excluir EAC induzido por cocaína.

Em nosso caso clínico, evidência de EAC foi observada na cinecoronariografia, supradesnivelamento transitório do segmento ST e bloqueio cardíaco paroxístico. Nossa hipótese é que mecanismos subjacentes à rejeição mediada por anticorpos podem causar EAC: anticorpos específicos do doador podem iniciar a cascata do complemento no endotélio do aloenxerto e causar lesão tecidual através de vias inflamatórias. As frações do complemento são depositadas na microvasculatura do aloenxerto resultando em um processo inflamatório caracterizado por ativação de células endoteliais, infiltração de macrófagos, upregulação de citocinas, aumento da permeabilidade vascular e trombose microvascular.^2^ Esse estado de disfunção endotelial, inflamação e hiperreatividade poderia precipitar episódios de EAC no contexto de rejeição aguda.

Casos clínicos de EAC apresentando arritmias malignas em pacientes transplantados cardíacos já foram relatados por Pistono et al.,^16^ e recentemente por M. Pagnoni et al.,^17^ entretanto, nesses dois casos não foram identificadas rejeição aguda celular ou humoral. Porém, a possível associação de EAC com rejeição aguda já foi considerada anteriormente em outros relatos de caso,^4 , 18^ os quais são consistentes com nosso caso clínico.

Conforme descrito anteriormente, o EAC pode ser uma manifestação precoce de DVE, o que não foi completamente excluído em nosso paciente, devido à baixa sensibilidade da cinecoronariografia para detecção de DVE em estágio inicial. Avanços recentes em imagens coronarianas invasivas, como ultrassonografia intravascular (USIV) e tomografia de coerência óptica (OCT, do inglês *optical coherence tomography* ), têm mostrado resultados promissores na detecção de DVE subangiográfica.^19^ Nesse contexto, a imagem intracoronária pode ter um papel significativo em pacientes cardíacos com EAC, ao excluir sinais precoces de DVE e, consequentemente, melhorar a estratificação de risco e a atenção aos pacientes.

Em nosso relato clínico, a rejeição celular e humoral se manifestou com EAC e arritmias malignas. Essas manifestações incomuns de rejeição aguda enfatizam que uma alta suspeita clínica precisa estar presente para sua pronta detecção e tratamento. Embora a etiologia do EAC ainda seja especulativa e provavelmente multifatorial, nosso caso clínico destaca a hipótese de que a hiperreatividade do endotélio e a inflamação causada pela rejeição aguda precipitaram o espasmo coronariano. Nosso caso clínico também mostra que a isquemia transitória causada pelo EAC pode precipitar distúrbios letais do ritmo. Mais estudos são necessários para entender completamente os mecanismos subjacentes ao EAC, sua relação com a rejeição do aloenxerto e o significado prognóstico.

## References

[1] McDonagh TA, Metra M, Adamo M, Gardner RS, Baumbach A, Böhm M, et al. ESC Scientific Document Group, 2021 ESC Guidelines for the diagnosis and treatment of acute and chronic heart failure, Eur Heart J.2021;42(36):3599-726. doi.org/10.1093/eurheartj/ehab368 10.1093/eurheartj/ehab368 34447992

[2] Kim NC, Youn JC, Kobashigawa JA. The past, present and future of heart transplantation. Korean Circ J.2018;48(7):565-90. 10.4070/kcj.2018.0189 PMC6031715 29968430

[3] Hamon D, Taleski J, Vaseghi M, Shivkumar K, Boyle NG. Arrhythmias in the heart transplant patient. Arrhythm Electrophysiol Rev.2014;3(3):149-55. doi:10.15420/aer.2014.3.3.149. 10.15420/aer.2014.3.3.149 PMC4711495 26835083

[4] Bisognano JD, Lindenfeld J, Hammond E, Zisman LS. Coronary artery vasospasm causing acute myocardial infarction in a heart transplant recipient. J Heart Lung Transplant. 2005;24(3):355-8. doi:10.1016/j.healun.2003.11.405 10.1016/j.healun.2003.11.405 15737767

[5] Barnard CN. The operation. A human cardiac transplant: an interim report of a successful operation performed at Groote Schuur Hospital, Cape Town. S Afr Med J. 1967 Dec 30;41(48):1271-4. 4170370

[6] Reitz BA, Bieber CP, Raney AA, Pennock JL, Jamieson SW, Oyer PE, et al. Orthotopic heart and combined heart and lung transplantation with cyclosporin-A immune suppression. Transplant Proc. 1981 Mar;13(1 Pt 1):393-6. PMID: 6791329 6791329

[7] Lund LH, Khush KK, Cherikh WS, Goldfarb S, Kucheryavaya AY, Levvey BJ, et al., et al. The registry of the International Society for Heart and Lung Transplantation: thirty-fourth Adult Heart Transplantation Report - 2017; focus theme: allograft ischemic time. J Heart Lung Transplant.2017;36:1037-46. 10.1016/j.healun.2017.07.016 28779893

[8] Rivinius R, Helmschrott M, Ruhparwar A, Erbel C, Gleissner CA, Darche FF, et al. The influence of surgical technique on early posttransplant atrial fibrillation - comparison of biatrial, bicaval, and total orthotopic heart transplantation. Ther Clin Risk Manag. 2017;13:287-297. doi: 10.2147/TCRM.S126869. 10.2147/TCRM.S126869 PMC5352240 28331331

[9] Gullestad L, Ross H, Myers J, Hoang k, Hunt S, Stinson EB, et al. Importance of decreased heart rate in predicting transplant coronary artery disease. Clin Transplant. 1997 Dec;11(6):628-32. PMID: 9408698 9408698

[10] Cooper MM, Smith CR, Rose EA, Schneller SJ, Spotnitz HM. Permanent pacing following cardiac transplantation. J Thorac Cardiovasc Surg. 1992 Sep;104(3):812-6. 1513170

[11] Mallidi HR, Bates M. Pacemaker use following heart transplantation. Ochsner J. 2017 Spring;17(1):20-4. PMID: 28331443; PMC5349631 28331443

[12] Akgün NA, Çiftci O, Yılmaz KC, Karaçaglar E, Aydinaep A, Sezgin A, et al. Prevalence and angiographic characteristics of coronary vasospasm detected at surveillance coronary angiograms among patients with heart transplants. Exp Clin Transplant. 2018 Mar;16(Suppl 1):85-8. doi: 10.6002/ect.TOND-TDTD2017.O34. 10.6002/ect.TOND-TDTD2017.O34 29527999

[13] Boffa GM, Livi U, Grassi G, Casarotto D, Isabella G, Cardaioli P, et al. Angiographic presentation of coronary artery spasm in heart transplant recipients. Int J Cardiol.2000;73(1):67-74. doi:10.1016/s0167-5273(99)00225-9. 10.1016/s0167-5273(99)00225-9 10748313

[14] Kobashigawa J. (ed) Clinical guide to heart transplantation. Los Angeles (CA): Springer; 2017. ISBN 978-3319437712

[15] Talarico GP, Crosta ML, Giannico MB, Summaria F, Calò L, Patrizi R. Cocaine and coronary artery diseases: a systematic review of the literature. J Cardiovasc Med (Hagerstown). 2017;18(5):291-4. doi:10.2459/JCM.0000000000000511 10.2459/JCM.0000000000000511 28306693

[16] Pistono M, Brentana L, Gnemmi M, Imparato A, Temporelli PL, Zingarelli E, et al. Early right coronary vasospasm presenting with malignant arrhythmias in a heart transplantation recipient without allograft vasculopathy. Int J Cardiol.2009;131(3):e120-e123. 10.1016/j.ijcard.2007.07.078. 17950482

[17] Pagnoni M, Regamey J, Adjedj J, Rogati G, Muller O, Tozzi P. Case report - coronary vasospasm in transplanted heart: a puzzling phenomenon. BMC Cardiovasc Disord. 2019;19(1):305. doi:10.1186/s12872-019-01280-8 10.1186/s12872-019-01280-8 PMC6924038 31856732

[18] Hruban RH, Kasper EK, Gaudin PB, Baughman KL, Baumgarter WA, Reitz BA, et al. Severe lymphocytic endothelialitis associated with coronary artery spasm in a heart transplant recipient. J Heart Lung Transplant. 1992;11(1 Pt 1):42-7. PMID: 1540611 1540611

[19] Guddeti RR, Matsuo Y, Matsuzawa Y, Aoki T, Lerman LO, Kushwaha SS, et al. Clinical implications of intracoronary imaging in cardiac allograft vasculopathy. Circ Cardiovasc Imaging. 2015;8(1):e002636. doi:10.1161/CIRCIMAGING.114.002636 10.1161/CIRCIMAGING.114.002636 25596140

