# Supplementary material for: Uma Manifestação Pouco Comum de Rejeição
Source: Arq Bras Cardiol. 2022 May 4;118(5):996–9. [Article in Portuguese] doi: 10.36660/abc.20210671 (PMC9368876; doi:10.36660/abc.20210671)
Supplement: Translation

# Translation: An Unusual Manifestation of Rejection

**Authors:** Carlos Xavier Correia de Resende, Pedro Grilo Diogo, Sandra Amorim, Gonçalo Pestana, José Pinheiro Torres, Filipe Macedo

**Affiliations:** 1 Portugal Centro Hospitalar Universitário de São Joao, Porto – Portugal

**Keywords:** Heart Failure, Heart Transplantation, Research, Graft Rejection, Diagnostic Imaging, Drug Therapy, Heart Block, Arrhythmias Cardiac, Coronary Vasospasm

## Introduction

Orthotopic heart transplantation is the current treatment of choice for selected patients with end-stage heart failure.^1^ With the improvement of surgical techniques and the efficiency of new immunosuppressive treatments, short-term survival has markedly improved throughout the years.^2^ However, these patients still suffer from important comorbidities caused by chronic transplant complications such as rejection, coronary allograft vasculopathy (CAV) and malignancy. Rhythm disorders are common in heart transplant patients and in some circumstances, they can be the first clinical manifestation of rejection.^3^ Coronary vasospasm has been recently associated with acute rejection and CAV,^4^ but the mechanisms underlying this phenomenon are still speculative.

We present a clinical case of acute heart transplant rejection, manifested by coronary vasospasm and advanced rhythm disorder.

### Case presentation

A 55-year-old male patient with end-stage ischemic heart failure underwent orthotopic heart transplantation in March 2019. The first-year follow-up endomyocardial biopsy (EMB) showed mild 1R cellular rejection (ISHLT) in 3 samples and moderate rejection (2R) in two samples, treated with increased doses of oral corticosteroids. At his last ambulatory appointment, the patient showed normal left ventricular function, with no humoral or cellular rejection identified in the last EMB (performed 4 months before admission). His blood samples showed infra-therapeutic levels of cyclosporine (94.2 ng/mL), leading to an increase in the ambulatory dose. He was also medicated with mycophenolate mofetil (MMF) (2g/day), prednisolone (5mg), atorvastatin, aspirin, cotrimoxazol and oral anti-diabetic drugs.

One year and five months after the transplantation, he was admitted to our emergency department with sudden altered state of consciousness. He showed a Glasgow Coma Score of 11 and was hemodynamically stable. On physical examination, the EKG, brain computed tomography and TTE were unremarkable. Blood samples revealed mild anemia (10 g/dL) and infra-therapeutic levels of cyclosporine (92 ng/mL), but no elevation of inflammatory markers; the toxicology was negative for alcohol or drugs of abuse. The patient did not collaborate during the electroencephalogram and due to his marked psychomotor agitation and confusion he was sedated and intubated. A lumbar puncture was performed, with no signs of infection in the cerebrospinal fluid. Cyclosporine was replaced by tacrolimus, considering the possibility of posterior reversible encephalopathy syndrome.

The patient was admitted to the intensive care unit, and suffered sudden cardiac arrest on the second day of hospitalization, with return of spontaneous circulation (ROSC) after two advanced life support cycles. The electrocardiogram after the ROSC ( Figure 1B ) documented ST-segment elevation in V2-V5. A second EKG 10 minutes after the event was normal ( Figure 1C ). After analysis of telemetry monitoring, complete heart block was identified before cardiac arrest ( Figure 1A ). On the same day, a second episode of complete heart block was documented with hemodynamic deterioration and a temporary pacemaker was implanted.

**Figure 1:** – A) Third-degree atrioventricular block before cardiac arrest; B) EKG after ROSC showing supra ST-elevation in V2-V5, isolated extra systole; C) Normal EKG 10 minutes after the event.

Coronary angiography was performed on the following day, showing markedly diffuse vasospasm in the left anterior descending and circumflex arteries, which resolved with intracoronary nitrate administration ( Figure 2A and B ). Moderate stenosis was identified in the proximal left anterior descending and first diagonal arteries. An endomyocardial biopsy was also performed.

**Figure 2:** – A) Coronary angiography showing markedly diffuse vasospasm of left anterior descending and circumflex arteries. B) Complete vasospasm resolution after nitrate intake.

On the subsequent days, a new episode of cardiac arrest was preceded by ventricular tachycardia, again with documentation of transient ST-segment elevation changes after ROSC ( Figure 3A ). Sporadic temporary pacemaker stimulation was also documented ( Figure 3B ). Given the suspicion of coronary vasospasm episodes, calcium channel blockers (CCB) and nitrate administration was initiated.

**Figure 3:** – A) Transient ST-T changes; B) Sporadic temporary pacemaker stimulation.

The endomyocardial biopsy results showed moderate 2R cellular rejection (ISHLT) and C4d positive humoral rejection with subsequent identification of donor specific anti-HLA class II antibodies (HLA-DR53). The patient was therefore treated with intravenous methylprednisolone pulses and remained clinically stable. With immunosuppressive treatment intensification, CCB and nitrate administration, the patient was hemodynamically stable with no new rhythm or EKG sudden changes. The diagnosis of acute allograft rejection causing coronary vasospasm and advanced rhythm disorders was presumed.

Two weeks after treatment, the endomyocardial biopsy was repeated, showing mild 1R cellular rejection (ISHLT) and no signs of humoral rejection. Due to the clinical and histological response, no additional pharmacological treatment was initiated. A cardioverter defibrillator was implanted, without complications.

During hospitalization, the patient suffered multiple nosocomial infections, requiring mechanical ventilation in two different occasions due to severe pneumonia. Because of the severe myopathy caused by prolonged hospitalization, he was discharged to a rehabilitation center and remains stable.

## Discussion

Since the first human heart transplantation performed by Dr. Christian Barnard in December 1967,^5^ several improvements have been observed throughout the years in relation to procedural and particularly postoperative management. The introduction of immunosuppression therapy in 1980 was a landmark, allowing a significant improvement in early survival rates.^6^ However, rejection remains one of the major causes of death after transplantation.^7^ Because the symptoms of rejection are often nonspecific, high clinical suspicious is paramount for prompt detection and treatment. In this context, serial EMBs remain the cornerstone for rejection diagnosis.

Rhythm disorders are common in heart transplant patients. However, with the worldwide implementation of the bicaval approach, which preserves the right atrium and the sinus node, the number of postoperative bradyarrhythmia events requiring permanent pacemaker implantation were significantly reduced.^8^ Bradycardia presenting late after heart transplantation has been associated with episodes of acute rejection^9^ and CAV, with the latter possibly leading to sinus node ischemia.^10^ This highlights the importance of recalling that in patients presenting with late-onset bradycardia or heart block after transplantation, the possibility of acute rejection and CAV must be investigated through echocardiography, EMB and coronary angiography.^11^ In these circumstances, pacemaker implantation is insufficient for an effective treatment until the underlying bradyarrhythmia cause can be treated.

Coronary artery spasm (CAS) is an underdiagnosed disease, particularly in heart transplant patients. This condition was considered to be rare; however, recent evidence shows a significant prevalence of CAS in routine coronary angiography in these patients.^12^ Boffa et al.^13^ documented coronary vasospasm in 12 patients (5% of the study population) after heart transplantation over a 5-year follow-up. During the follow-up, 80% of the patients developed organic stenosis and 50% of those with multiple vasospasms died. These data highlights the hypothesis that CAS after heart transplantation could be a marker of poor prognosis and early manifestation of CAV.

The etiology and pathophysiology of CAS in heart transplant patients remains speculative, but several mechanisms have been reported: abnormal autonomic nervous system, endothelial dysfunction, coronary smooth muscle hyperactivity, and perivascular component inflammation.^14^ One of the presumed causes of CAS is cocaine consumption, due to the direct adrenergic stimulation of coronary arteries.^15^ Particularly in the context of CAS episodes that present early after transplantation, a thorough medical history of the heart donor and recipient could help to exclude cocaine-induced CAS.

In our clinical case, evidence of CAS was seen in the coronary angiography, transient ST-segment elevation and paroxysmal heart block. We hypothesize that mechanisms underlying antibody-mediated rejection can cause CAS: donor-specific antibodies may initiate the complement cascade in the allograft endothelium and cause tissue injury via inflammatory pathways. Complement fractions are deposited in the allograft microvasculature, resulting in an inflammatory process characterized by endothelial cell activation, macrophage infiltration, cytokine upregulation, increased vascular permeability and microvascular thrombosis.^2^ This state of endothelial dysfunction, inflammation and hyperreactivity could precipitate CAS episodes in the context of acute rejection.

Clinical cases of CAS presenting with malignant arrhythmias in heart transplantation patients have been reported by M. Pistono et al.^16^ and recently by M. Pagnoni et al.^17^ ; however, in these two cases no cellular or humoral acute rejection were identified. Nevertheless, the possible association of CAS with acute rejection has been previously considered in other case reports,^4 , 18^ which are consistent with our clinical case.

As previously described, CAS could be an early manifestation of CAV, which was not completely ruled out in our patient, due the low sensitivity of coronary angiography for detecting early-stage CAV. Recent advances in invasive coronary imaging, such as intravascular ultrasound (IVUS) and optical coherence tomography (OCT) have shown promising results in detecting subangiographic CAV.^19^ In this context, intracoronary imaging could have a significant role in the diagnosis of CAS in heart patients by excluding early signs of CAV and, consequently, improving risk stratification and patient surveillance.

In our clinical report, cellular and humoral rejection manifested as CAS and malignant arrythmias. These unusual manifestations of acute rejection emphasizes that high clinical suspicion needs to be present for its prompt detection and treatment. Although the etiology of CAS is still speculative and probably multifactorial, our clinical case highlights the hypothesis that endothelium hyperreactivity and inflammation caused by acute rejection precipitated the coronary spasm. Our clinical case also shows that transient ischemia caused by CAS can precipitate deadly rhythm disorders. Further studies are needed to fully understand the mechanisms underlying CAS, their relationship with allograft rejection and prognostic significance.
